# Reproducibility of point-of-care cardiac ultrasound in Chagas disease by a non-cardiologist with limited training

**DOI:** 10.1186/2036-7902-6-S1-A18

**Published:** 2014-01-31

**Authors:** Lucas Jordan Kreuser, Maria Carmo P Nunes, Robert Reardon, Antonio L Ribeiro

**Affiliations:** 1 University of Minnesota Medical School, Minneapolis, MN, USA

## Background

Chagas disease is a major cause of heart failure in Latin America. Many Chagas disease patients live in rural areas under poor economic conditions with limited access to formal echocardiography. Point-of-care ultrasound is rapidly gaining recognition as a powerful diagnostic tool in such fields as emergency medicine, intensive care, and primary care, but is not commonly utilized in Chagas-endemic regions. In this study we sought to determine the reproducibility of point-of-care cardiac ultrasound performed by a non-cardiologist in Chagas patients.

## Methods

Consecutive patients with Chagas disease receiving regularly-scheduled echocardiograms were recruited at a tertiary care teaching hospital in Brazil. A board-certified cardiologist performed and interpreted an echocardiogram with a conventional full function echocardiography machine (iE33 Philips Medical Systems), and a 4^th^-year medical student performed and interpreted a cardiac exam with a portable ultrasound machine (Sonosite MicroMaxx). The medical student was blind to clinical and the cardiologist’s echocardiographic data. A range of quantitative and visual estimation data were collected and compared between the two exams.

## Results

A total of 41 patients (mean age=49.5) were examined, and 16 (39%) had at least mild LV systolic dysfunction per the cardiologist´s exam. Measurements from the two observers were highly correlated (ICC values for Long-axis LVDd=0.958 and LVEF=0.930). Qualitative visual estimation of LV systolic function was highly reproducible (Kappa = 0.76, Observed agreement = 0.854), as was RV systolic function (Kappa = 0.55, Observed agreement 0.900).

## Conclusion

Point-of-care cardiac ultrasound performed and interpreted by a non-cardiologist with limited ultrasound training is accurate and reproducible for detecting cardiac dysfunction. Point-of-care cardiac ultrasound provides clinically valuable information in patients with Chagas disease and could help clinicians determine management of such patients in a wide variety of care settings.

